# Identification and Validation of Magnolol Biosynthesis Genes in *Magnolia officinalis*

**DOI:** 10.3390/molecules29030587

**Published:** 2024-01-25

**Authors:** Yue Yang, Zihe Li, Hang Zong, Shimeng Liu, Qiuhui Du, Hao Wu, Zhenzhu Li, Xiao Wang, Lihui Huang, Changlong Lai, Meide Zhang, Wen Wang, Xianqing Chen

**Affiliations:** 1 School of Ecology and Environment, Northwestern Polytechnical University, Xi’an 710072, China; yangyue@synbiolab.cn (Y.Y.); lzhleo@mail.nwpu.edu.cn (Z.L.); zonghangnb@163.com (H.Z.); lizhenzhu@synbiolab.cn (Z.L.); 2 Jiaxing Synbiolab Biotechnology Co., Ltd., Jiaxing 314006, China; liushimeng@synbiolab.cn (S.L.); duqiuhui@synbiolab.cn (Q.D.); wuhao@synbiolab.cn (H.W.); wangxiao@synbiolab.cn (X.W.); huanglihui@synbiolab.cn (L.H.); laichanglong@synbiolab.cn (C.L.); 3 Institute of Chinese Herbal Medicines, Hubei Academy of Agricultural Sciences, Enshi 445000, China; emailtoecho@163.com

**Keywords:** *Magnolia officinalis*, transcripts, in vitro, magnolol synthesis, enzyme activity

## Abstract

Bacterial infections pose a significant risk to human health. Magnolol, derived from *Magnolia officinalis*, exhibits potent antibacterial properties. Synthetic biology offers a promising approach to manufacture such natural compounds. However, the plant-based biosynthesis of magnolol remains obscure, and the lack of identification of critical genes hampers its synthetic production. In this study, we have proposed a one-step conversion of magnolol from chavicol using laccase. After leveraging 20 transcriptomes from diverse parts of *M. officinalis*, transcripts were assembled, enriching genome annotation. Upon integrating this dataset with current genomic information, we could identify 30 laccase enzymes. From two potential gene clusters associated with magnolol production, highly expressed genes were subjected to functional analysis. In vitro experiments confirmed MoLAC14 as a pivotal enzyme in magnolol synthesis. Improvements in the thermal stability of MoLAC14 were achieved through selective mutations, where E345P, G377P, H347F, E346C, and E346F notably enhanced stability. By conducting alanine scanning, the essential residues in MoLAC14 were identified, and the L532A mutation further boosted magnolol production to an unprecedented level of 148.83 mg/L. Our findings not only elucidated the key enzymes for chavicol to magnolol conversion, but also laid the groundwork for synthetic biology-driven magnolol production, thereby providing valuable insights into *M. officinalis* biology and comparative plant science.

## 1. Introduction

Antibiotic resistance represents a pressing global health concern, impacting food security and development by complicating the treatment of infections with conventional antibiotics [1,2,3]. Natural plant-derived compounds have exhibited significant antimicrobial activity, especially magnolol, a small molecule extracted from various parts of *Magnolia officinalis*, such as the dried bark, root bark, and branch bark [4,5,6]. Notably, magnolol has demonstrated an inhibitory activity against methicillin-resistant *Staphylococcus aureus* (MRSA) and methicillin-susceptible *S. aureus* [7], thereby showcasing its comparable bactericidal activity to fluconazole [8,9].

The current methods for obtaining magnolol involve either direct extraction from the dry bark of *M. officinalis* or extraction of high-purity magnolol from its bark [10,11,12]. Nevertheless, two main challenges impede these practices: the lengthy cultivation time of 10–15 years that is required for *M. officinalis* and the low concentration of magnolol in *M. officinalis*, which hovers around 1% [13,14,15].

Efforts to efficiently produce magnolol have involved chemical synthesis, dating back to the 1950s when Runeberg proposed using chavicol as a substrate for magnolol synthesis through suitable chemical transformation [16]. In the 1980s, Clark et al. proposed synthesizing magnolol from phenol; however, the reaction was not specific, which produced a low yield and the formation of other by-products [17]. Moreover, the high degree of consumption of organic solvents in the process added to the environmental pollution, making it unsuitable for industrial production [18]. In addition, the high energy required for solvent recycling and the multistage separation processes further limit its applicability [19]. Therefore, developing techniques for the efficient and environmentally friendly production of magnolol is imperative.

Advances in synthetic biology and molecular biology have revolutionized our understanding of biological systems and their potential applications. For instance, the synthesis of artemisinic acid, a precursor to artemisinin, using *Saccharomyces cerevisiae*, has significantly enhanced resource efficiency and cost-effectiveness [20]. Synthetic biology has also facilitated the production of cannabinoids, dencichine, breviscapine, and amino acids [21,22,23]. However, applying synthetic biology methods for magnolol synthesis remains challenging owing to the limited understanding of the biosynthesis pathway in *M. officinalis*, although it is speculated to stem from the common lignan biosynthesis pathway [9,24].

Through comprehensive chemical and biochemical analysis, we hypothesize that magnolol is synthesized by linking two chavicol molecules catalyzed by the enzyme laccase. Laccase plays a vital role in oxidizing aromatic substrates, primary phenolics, and diamines or benzenethiols, generating water as a byproduct [25,26]. In nature, laccases exhibit diverse functions in both anabolic and catabolic processes. Notably, in the anabolic processes, laccases oxidize low-molecular-weight phenolics into dimeric products, which undergo further reactions to form trimers, oligomers, and polymers through self/cross-coupling. Consequently, laccase-mediated biosynthesis represents an effective biotechnological tool that is applicable across industries. Thus, employing laccase to link two chavicol molecules for magnolol production in *M. officinalis* seems plausible.

Our study initially involved transcriptome sequencing and RNA-seq reads assembly from different *M. officinalis* tissues to validate our hypothesized magnolol pathway. Leveraging RNA-seq data, we refined the genome annotation of *M. officinalis* by annotating transcripts, categorizing their biological functions and pathways, and eventually identifying all laccase genes in *M. officinalis*. Based on the expressions and gene-clustering genomic structure features, we selected highly expressed laccase genes in the tissues to examine their ability to synthesize magnolol in vitro. Magnolol catalyzed by laccase was confirmed through high-performance liquid chromatography (HPLC) and mass spectrometry (MS), which subsequently pinpointed the laccase gene responsible for magnolol synthesis. Following the identification, we opted for the engineering of the laccase enzyme to enhance its thermal stability and enzymatic activity, concurrently elucidating the underlying mechanism. Overall, our findings provide compelling evidence for synthesizing magnolol from chavicol using laccase as a catalyst, underscoring the potential of synthetic biology and molecular biology in producing valuable compounds with mild reaction conditions and high specificity.

## 2. Results

### 2.1. Biosynthetic Pathway for Magnolol and RNA Sequencing of M. officinalis Tissues

**Figure 1:**
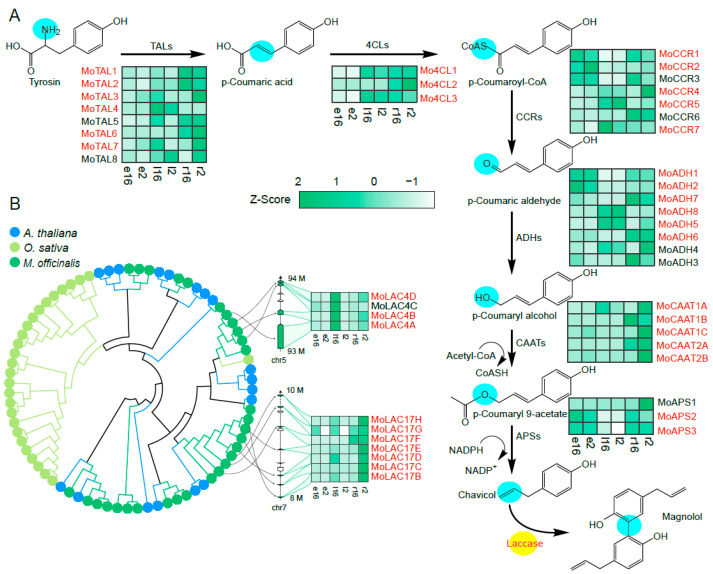
Identification and expression of magnolol-related genes in *M. officinalis*. (**A**) The magnolol pathway and the potential applications of laccases. Different copies of genes related to the magnolol pathway, including TALs, 4CLs, CCRs, ADHs, CAATs, and APSs, can be observed. Genes in red font are differentially expressed genes in at least one tissue. (**B**) LAC gene family gene tree and two tandem gene clusters’ chromosomal locations and structures. The chemical structure marked with a blue circular label indicates the key site for enzymatic catalysis. On the gene tree, the yellow lines represent genes from the *O. sativa*, the blue lines represent genes from *A. thaliana*, the green lines represent genes from *M. officinalis*, and the black lines indicate genes from different species that cluster under a single clade. Within the gene cluster, green arrows represent different laccase lengths, while white arrows denote other enzymes within the gene cluster. The expression values of the genes were converted to the Z-scores, and the graph displays the results pooled for two individuals of *M. officinalis* with ages of 2 and 16 years, respectively, and three types of tissue samples: root (r), leaf (l), and bark (e).

Magnolol is an important medicinal compound. Although it was previously postulated that magnolol is biosynthesized from matairesinol, the structural disparity between these two compounds suggests that numerous enzymatic steps may be involved in converting matairesinol to magnolol. Based on the previous reports [27,28,29,30], we have proposed the existence of the chavicol synthesis pathway *M. officinalis* (Figure 1A). This pathway likely commences with tyrosine catalysis by enzymes such as tyrosine ammonia-lyase (TAL), 4-coumarate CoA ligase (4CL), cinnamoyl-CoA reductase (CCR), and alcohol dehydrogenase (ADH), to yield *p*-coumaryl alcohol. Subsequent enzymatic action involving coniferyl alcohol acetyltransferase (CAAT) and allylphenol synthases (APS) convert *p*-coumaryl alcohol to chavicol. Our hypothesis suggests that magnolol is then synthesized from the precursor chavicol catalyzed by laccase. To validate this proposed magnolol biosynthetic pathway in *M. officinalis*, we conducted transcriptome sequencing of magnolol-producing tissues.

Four or three replicates of 16-year-old bark and leaves were obtained from these samples, and three replicates for other tissues, yielding an average of 6.6 Gb of data per sequenced sample (Table S1), with an average mapping rate exceeding 80% and an average Q30 read quality rate surpassing 97%. The assembled RNA-seq reads were mapped to a previously reported *M. officinalis* genome [31], revealing 19,946 protein-coding genes expressed in at least one tissue (transcripts per million, TPM ≥ 1, Table S2). The mean length of the CDS stands at 1224 bp. The total count of mRNA identified amounts to 52,692. With respect to the completeness of the proteome, we observed a BUSCO score of 79.3%, while the genome’s BUSCO score reaches 86.2%, indicating a substantial representation of conserved genes.

Principal component analysis (PCA) illustrated greater variation between tissues than between ages (Figure S1). The differential expression patterns across various tissues are delineated in Table S3 and Figure 1. Our investigation conducted differential gene expression across various tissues, indicating that genes annotated in red font (Figure 1A) represent those exhibiting differential expression in at least one tissue type. Notably, the genes associated with magnolol precursor, chavicol, and metabolism predominantly displayed higher expressions in the root and leaf or bark tissues. In addition, gene ontology (GO) enrichment analysis was conducted on the annotated genes, with the outcomes of this analysis across molecular function, cellular component, and biological process, as displayed in Figures S2, S3 and S4, respectively.

Transcriptome analysis identified 30 potential laccase genes in *M. officinalis* (Figure S5). The comparative genomics of the laccase gene family within *M. officinalis* in contrast to *Arabidopsis thaliana* and other proximate taxa demonstrates a pronounced expansion of the laccase gene repertoire in *M. officinalis* (Figure 1B and Figure S6). This expansion is predominantly attributed to events of gene tandem duplication. Notably, two gene clusters, MoLAC4 and MoLAC17, were identified as potential contributors to magnolol biosynthesis (Figure 1B), which is consistent with the role of biosynthetic gene clusters in the evolution of pathways for secondary metabolites [32]. Furthermore, we examined the spatiotemporal expressions of the laccase genes and noted high expression in the roots and leaves (Figure S5).

To identify the potential catalytic genes for magnolol synthesis from chavicol, we selected highly expressed laccase in the roots, leaves, and bark, including *MoSKU5F*, *MoLAC7B*, and *MoLAC14*, along with those in the MoLAC4 and MoLAC17 gene clusters, including *MoLAC4A*, *MoLAC4B*, and *MoLAC17F*. Subsequently, these six genes were tested as potential contributors to the magnolol synthesis pathway.

### 2.2. Laccase-Catalyzed Magnolol Synthesis

**Figure 2:**
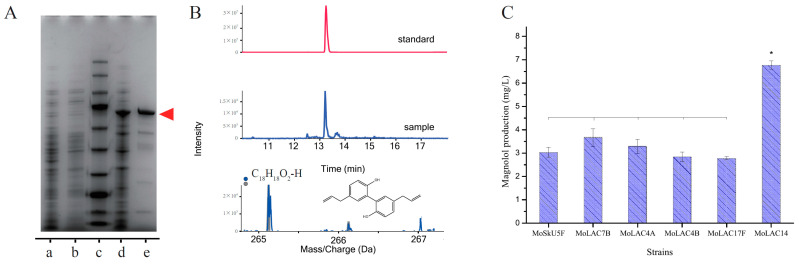
Verification of the laccase protein expression and the identification of magnolol products. (**A**) Sodium dodecyl sulfate-polyacrylamide gel electrophoresis (SDS-PAGE) analysis of the expressed laccase proteins, with an expected molecular weight of approximately 70.1 kDa. Lanes show an empty pET28a plasmid control (a and b), molecular weight standards (c), the supernatant sample (d), and pure protein (e), as indicated by red arrows. (**B**) High-performance liquid chromatography (HPLC) and mass spectrometry (MS) for magnolol detection (RT = 13.2 min). In the mass spectrum, the gray dots and lines represent the theoretical mass spectral data, while the blue dots and lines correspond to the actual mass spectral data observed in the experiment. The molecular formula is C_18_H_18_O_2_. (**C**) Laccase screening based on magnolol synthesis. Statistical analysis was performed using the Duncan’s multiple range test, with a significance level at *p* < 0.05. * < 0.05. Bars denote the standard error of the mean.

The CDS sequences and amino acid sequences of the aforementioned genes were retrieved from the *M. officinalis* transcriptomes and genome (Supplementary Table S4), synthesized by Wuhan Genecreate Biotechnology Co., Ltd. (Wuhan, China). Then, the genes were integrated into the pET-28a vector between the Nde I and Xho I sites by Gibson assembly, respectively (Table S5). Then, the constructed plasmids were chemically transformed into the BL21(DE3) *E. coli* strain, generating an expression strain for each gene (Table S6). Each strain was cultured at 37 °C, and the cloned gene expression was induced by isopropyl β-D-1-thiogalactopyranoside (IPTG), with the concurrent addition of Copper (II) ions (Cu^2+^) to assist protein folding. After high-pressure homogenization, the cell lysate was purified using a nickel column to obtain a pure protein solution. SDS-PAGE analysis confirmed the protein sizes (Figure 2A). The catalytic activity was assessed by quantifying the production of magnolol under uniform reaction conditions.

The identification of the product through HPLC and MS (Figure 2B) revealed a liquid phase peak aligning well with the standard peak at 13.2 min, with a mass spectral molecular weight matching that of standard magnolol (Solarbio). All six of the selected laccases demonstrated the capacity to catalyze magnolol synthesis from chavicol (Figure 2C). Among the six genes, MoLAC14 exhibited the highest activity and expression in the leaves (Figure S5). Preliminary detection indicated a production content of 7 mg/L under the reaction conditions for both enzyme and substrate addition. MoLAC7B exhibited the second-highest catalytic activity. These findings affirm that laccases can synthesize magnolol from chavicol.

Intriguingly, our exploration within synthetic biology led to the detection of a byproduct. Mass spectrometric analysis suggests its molecular formula to be C_27_H_26_O_3_ (Figure S7), with a corresponding retention time of 13.8 min. This molecule is presumed to be a trimer, potentially identified as Dunnianol or an isomeric variant, likely originating from the parent compound chavicol. Furthermore, genes associated with chavicol synthesis exhibited a high expression in the *M. officinalis* transcriptome. Upon heterologous expression of CAAT and APS from *M. officinalis* to catalyze *p*-coumaric aldehyde and produce chavicol, the final chavicol yield reached 0.04 mg/L.

### 2.3. Optimization of Magnolol Synthesis through the MolAC14 Enzyme

Our results revealed that MoLAC14 laccase is the highest magnolol yield producer. Further optimization of the reaction conditions was performed to enhance yields. We assessed the impacts of substrate addition, reaction time, and reaction temperature on the MoLAC14 enzyme activity by maintaining certain fixed parameters and optimizing others individually. By establishing a 50 mM phosphate buffer system at pH 7.5 with an enzyme concentration of 0.5 mg/mL, we ceased the reaction by heating the system to 100 °C for 10 min. It is therefore of paramount importance to maintain the equilibrium between the substrate concentration and the magnolol yield of MoLAC14 enzyme. Excessive substrate concentrations may inhibit the enzyme, reducing conversion rates, while lower concentrations could impede catalysis due to the enzyme’s high-affinity constant [33,34,35].

**Figure 3:**
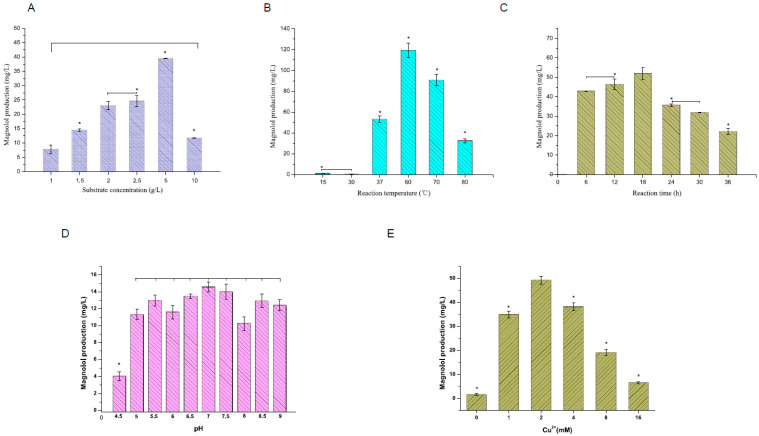
The influence of the reaction parameters on laccase catalytic activity. (**A**) Relationship between substrate concentration and magnolol production. (**B**) The impact of reaction duration on magnolol synthesis. (**C**) Temperature dependency of magnolol production. (**D**) Influence of pH on magnolol production. (**E**) The role of copper ion (Cu^2+^) concentration in magnolol yield. Statistical analysis was performed using Duncan’s multiple range test, with a significance level at *p* < 0.05. * < 0.05. Bars denote the standard error of the mean.

To investigate the substrate concentration’s effect on the reaction system, we kept the reaction temperature and time constant at 60 °C and 3 h, respectively, while varying the substrate concentration from 1 to 10 g/L. Figure 3A illustrates the influence of different substrate amounts on magnolol production. The highest magnolol yield, at 38 mg·L^−1^, occurred at a 5 g/L substrate concentration catalyzed by the MoLAC14 enzyme.

The reaction time is pivotal in enzyme assays, as it allows sufficient time for detectable product formation [36]. We examined the impact of extending reaction times, ranging from 6 to 36 h, on MoLAC14’s product yield. These reactions, conducted at a constant temperature of 60 °C with a constant substrate concentration of 1 g/L, maintained a copper (II) ion concentration of 1 mM. Incomplete reactions yielded lower product levels than fully reacted samples. The optimal product yield, reaching 50 mg/L, occurred at an 18 h reaction time (Figure 3B). You et al. [37] and Nicolás et al. [38] suggested the potential decomposition of magnolol over prolonged periods, thus rendering 18 h the most suitable reaction time for this assay.

The temperature significantly affects the MolAC14 activity, which influences the magnolol conversion rates. Determining the optimal temperature for MolAC14 activity entailed employing a substrate concentration of 1 g/L and a reaction time of 3 h, while maintaining a copper (II) ion concentration at 1 mM. Notably, temperature adjustments (raising the temperature can increase the reaction rate) affect enzyme catalysis by altering the activation energy and enzyme structures (high temperatures can lead to enzyme denaturation and loss of activity) [39,40]. Our findings indicated 60 °C as the optimal reaction temperature for MolAC14, yielding 120.3 mg/L of magnolol. The product amount remained <50 mg/L when the temperature was <60 °C. Conversely, the product amount gradually decreased as the temperature exceeded 60 °C (Figure 3C).

In addition, laccase, a Cu^2+^-dependent enzyme, exhibits pH sensitivity significantly influenced by Cu^2+^ concentration [41,42]. Our study extensively explored pH and Cu^2+^ concentration effects on laccase enzymatic activity. With a substrate concentration of 1 g/L, a reaction time of 3 h, and a constant temperature of 60 °C, MoLAC14 demonstrated catalysis of chavicol to magnolol across a broad pH range from 5–9 (Figure 3D). The optimal catalytic activity occurred at pH 7–7.5, yielding 14.5 mg/L magnolol. In the absence of added Cu^2+^, MoLAC14 exhibited minimal activity, with only trace magnolol production. Magnolol yields progressively increased with higher Cu^2+^ concentration, peaking at 49 mg/L with 2 mM Cu^2+^ (Figure 3E). Further elevating copper ion concentration reduced the MoLAC14 catalytic activity.

### 2.4. Modification of MoLAC14 Enzyme

Laccase demonstrates an optimal reaction temperature of 60 °C, which is distinctively higher than the typical enzymatic optimum temperature of ambient or 37 °C, showcasing unique thermotolerance abilities [43,44]. To improve its practical longevity, the focus centered on engineering the enzyme for improved thermal stability. To achieve this point, we assessed enzyme activity post-heat treatment to measure residual mutations’ impact. MoLAC14 heated at 60 °C for varying durations showed no activity loss after 3 h. However, longer heating times led to minor activity reductions, dropping below 50% after 24 h. Overall, MoLAC14 displayed robust thermal stability, exhibiting a 60 °C half-life of 24 h for 50% activity loss (Figure S8A). Thus, we assessed stability after a 24 h, 60 °C treatment.

**Figure 4:**
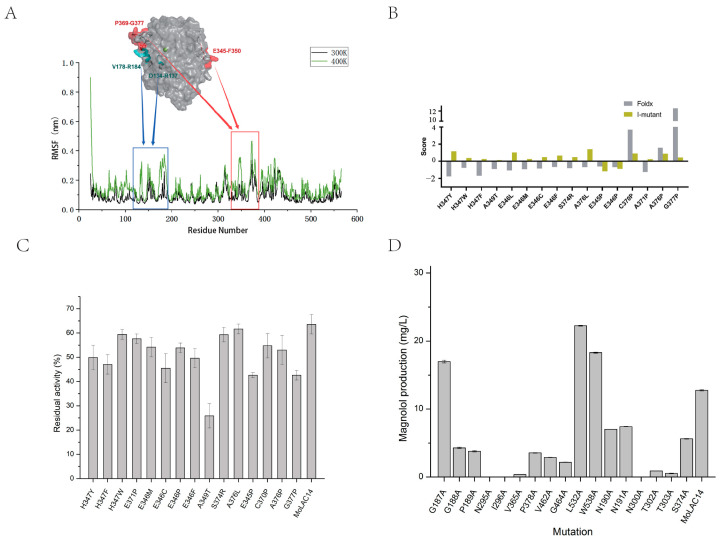
Modifications implemented on MoLAC14 to increase its thermal stability and the identification of amino acid residues crucial to thermal stability. (**A**) The flexible surface residues within MoLAC14’s loop structures. (**B**) Free energies of potential mutation sites computed using FoldX and I-Mutant2.0 algorithms. (**C**) The variance in thermal stability due to different mutants of MoLAC14. (**D**) The influence of different mutations on the catalytic activity of MoLAC14. Bars denote standard error of the mean.

**Figure 5:**
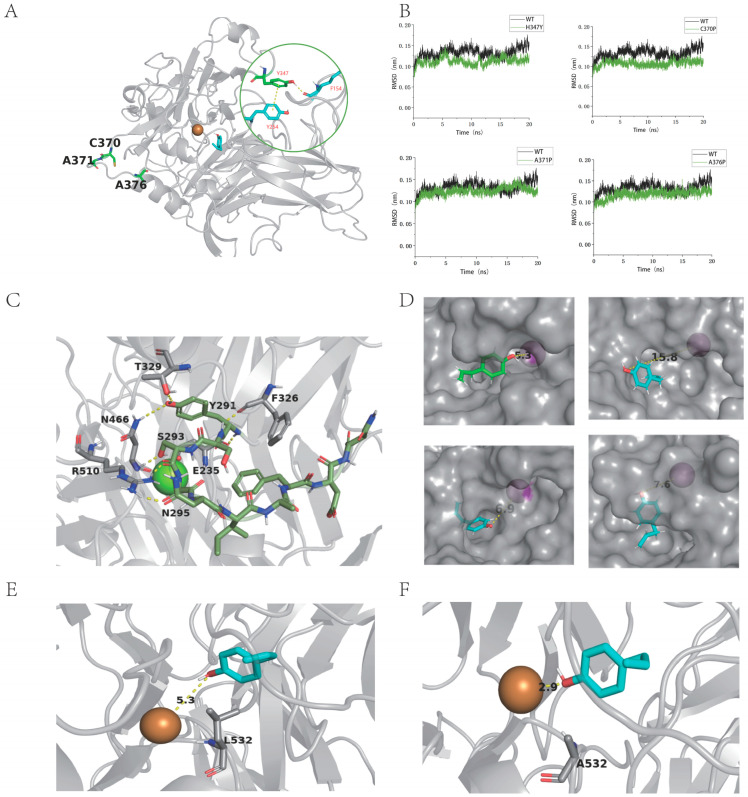
Mechanisms of the key residues impacting the thermal stability and catalytic activity of MoLAC14. (**A**) Residues located on the surface loop of the protein. (**B**) RMSD analysis of the four mutant variants. (**C**) The network of hydrogen bonds with surrounding residues. (**D**) Distances at distinct contact points between the substrate and copper ions pre- and post-mutation of residues N295, I296, N300, T302, and T303. (**E**) The intermolecular distance between piperonyl alcohol and Cu^2+^ of MoLAC14. (**F**) The intermolecular distance between piperonyl alcohol and Cu^2+^ of the L532 mutant.

As guided by the MoLAC14 structural modeling and substrate docking, we selected flexible surface residues within loops for virtual saturation mutagenesis (Figure 4A). Calculating free energies of mutants using FoldX and I-Mutant 2.0 identified critical sites for mutation, including H347Y, H347F, H347W, E371P, E346L, E346M, E346C, E346P, E346F, A349T, S374R, A376L, E345P, C370P, A376P, and G377P (Figure 5B).

The A349T mutant exhibited significantly reduced residual activity, indicating the pivotal role of residue 349 in thermostability (Figure 5C). All 16 MoLAC14 mutants showcased measurable effects on thermostability, with particularly pronounced alterations observed in A349T, E345P, G377P, H347F, E346C, and E346F.

Laccase protein crystal structures revealed three Cu^2+^ binding sites, coordinating four Cu^2+^ ions. Based on spectroscopic features, these are classified as type 1 Cu^2+^ (blue), type 2 Cu^2+^ (normal), and type 3 binuclear Cu^2+^ (coupled). The catalytic mechanism of laccases begins with a single-electron transfer from the substrate to type 1 Cu^2+^. Upon entering the MoLAC14 active site, substrate chavicol binds to the type 1 Cu^2+^ and transfers an electron via the Cys-His pathway to the trinuclear copper cluster (TNC), where O_2_ is reduced by the Cu^2+^ electron to generate H_2_O. The type 1 mononuclear center determines laccase substrate selectivity, with the surrounding loop region controlling both substrate access to the binding pocket and type 1 Cu^2+^ electron transfer.

For identifying residues that significantly influenced magnolol synthesis, mutational analysis of the substrate binding pocket was conducted. Molecular docking and 20 ns molecular dynamics simulations first approximated the binding pocket location. Aligning the dynamic trajectories and performing an alanine scanning (Figure S8B) for residues situated within a proximity of 5 Å to the substrate affirmed the predictions posited through the docking process. We eventually selected 18 residues for further mutation and activity assays.

Experimental validation (Figure 4D) revealed that three mutants (i.e., N295A, I296A, and N300A) lost activity, two mutants (i.e., T302A, T303A) had substantially decreased activity, while the remaining mutants exhibited modest increases or decreases in their activity. The L532A mutant achieved magnolol yields of 22.26 mg/L, while the W538A and G187A mutants reached 18.3 mg/L and 16.99 mg/L magnolol, representing 74.44%, 43.38%, and 33.12% activity improvements over wild-type MoLAC14. When compared to MoLAC14, the other 15 mutants displayed reduced activity, potentially due to disrupted substrate binding, especially for mutants N295A, I296A, and N300A.

Using the L532A mutant to catalyze chavicol under optimized conditions, magnolol concentrations reached a remarkable 148.83 mg/L at 5 g/L substrate, representing the highest reported biological magnolol production until date.

The enzyme kinetics for both wild-type laccase and engineered laccase were determined (Table 1). The Km value of the L532A mutant is higher than that of MoLAC14, indicating a lower affinity between the enzyme and the substrate. The L532A mutant converts a greater number of substrates per unit time compared to MoLAC14. Notably, its Kcat is about three times greater than that of MoLAC14. Specifically, for the substrate chavicol, the Km/Kcat of the L532A mutant was significantly higher than that of MoLAC14. Accordingly, the production of magnolol in the L532A mutant (148.83 mg/L) was greater than that in MoLAC14 (120.3 mg/L).

### 2.5. Mechanism of the Impact on Thermal Stability and Activity by Critical Residues of MoLAC14 Mutants

Residues such as C370P, A371P, A376P, and H347Y, primarily located on the protein surface loop, differ from the catalytic cavity (Figure 5A). Mutations of C370P, A371P, and A376P to the rigid proline can stabilize their loop. Further analysis of the four mutants revealed decreased root-mean-square deviation (RMSD), indicating reduced structural fluctuations and overall protein stability (Figure 5B). The H347Y mutant formed a new π-π conjugated bond with the phenyl ring on Tyr254, and a new hydrogen bond was created with Phe154. These new bonds stabilize the loop where H347 resides, enhancing loop stability. The analysis of the enzyme’s three-dimensional structure suggests that these mutations, which significantly diminish enzymatic activity, are located on the same loop, verifying the loop’s importance in the substrate’s catalytic cavity. This finding further confirms the proposed substrate binding pocket location.

Examining the interactions between the loop and other residues indicates a dense network of hydrogen bonds (Figure 5C) maintains the stable conformation of the substrate binding pocket. The simulation results displayed a significant reduction in hydrogen bonds after mutating five sites—N295, I296, N300, T302, and T303—to alanine (Figure S8B). This variation disrupted the loop stability, preventing a stable conformation in the enzyme’s catalytic pocket, and altering the substrate’s positioning away from the active center. This increased distance between the substrate and copper ions, from 5.3 Å to 15.8 Å, 6.9 Å, and 7.6 Å, respectively (Figure 5D), which led to enzyme inactivation or a significant decrease in the activity.

The L532A mutant significantly enhanced the enzymatic activity, surpassing the wild-type by over 74%. To elucidate the reason for this increase, we constructed wild-type–piperonyl alcohol and mutant L532A-piperonyl alcohol complexes through molecular docking. Docking results of the wild-type suggested that leucine at position 532 hinders electron transmission from piperonyl alcohol to T1 Cu^2+^. Conversely, the alanine mutation reduced steric hindrance, easing piperonyl alcohol access to Cu^2+^. This reduces the intermolecular distance from 5.3 Å to 2.9 Å, thereby increasing the chance of electron transmission between piperonyl alcohol and Cu^2+^, resulting in enhanced activity (Figure 5E,F). Concurrent experimental results provide insight into substrate conformation, which allows for the potential redesigning of the binding pocket to enhance the substrate’s affinity and activity.

## 3. Discussion

Mohamad et al. reported that magnolol was synthesized chemically through aryl coupling and the methoxymethyl ether of 4-allyl-2-lithiophenol using the zinc chloride method, as well as from 5,5′-dibromo-2,2′-dimethoxybiphenyl by allylation with allyltributylstannane, followed by ether cleavage [45]. While a potential biosynthetic route for magnolol has been suggested previously, with relevant genes initially explored via bioinformatics [21,34], the complete biosynthesis pathway had not been experimentally validated until now [46]. Our current results show that laccase can catalyze the synthesis of magnolol from chavicol, thereby confirming the last step of this new biosynthesis pathway within the *M. officinalis* plant. Interestingly, all the genes involved in chavicol biosynthesis from tyrosine, including TAL, 4CL, CCR, ADH, CAAT, and APS, are present as multiple copies in the *M. officinalis* genome (Figures S6 and S9, Table S7). Moreover, we identified several genes involved in the biosynthesis of chavicol that are expressed in tissues where magnolol is produced, which ultimately resulted in the biosynthesis of 0.04 mg/L of chavicol. This finding marks the first report on the biosynthetic pathway of magnolol, which has been indirectly validated through our experimental approach. Furthermore, our results show that all six tested laccases in our experiments could catalyze the synthesis of magnolol, indicating the high possibility of *M. officials* using lacceses to produce magnolol. Our characterization of biosynthetic pathways revealed that the synthesis of magnolol necessitates the utilization of acetyl-CoA and the reducing power of NADPH. Recent studies have further indicated that Yarrowia lipolytica can supply a robust provision of acetyl-CoA precursors and ample reducing power [47,48], thus catering to the requisites of heterologous synthetic pathways. Previously, flavonoids were considered as low-yield metabolites with rate-limiting steps [49], primarily due to the limited availability of precursor acetyl-CoA and suboptimal metabolic pathway engineering, culminating in fermentative yields scarcely exceeding 1 g/L [21,50,51]. However, with the intervention of oleaginous yeast, metabolic yields for certain compounds that appeared challenging to surpass 1 g/L can now be effortlessly elevated to several grams per liter [52,53,54]. Our forthcoming endeavors will focus on integrating the chavicol biosynthetic pathway and the critical laccases identified within the engineered oleaginous yeast strains to enable de novo biosynthesis and high-yield production of magnolol. This approach may accelerate the realization of industrial-scale production of magnolol at an unprecedented pace. We identified a laccase, MoLAC14, with a high catalytic ability to catalyze two chavicols into a magnolol. This finding laid a simple and efficient foundation for producing magnolol through synthetic biology. Our data and analysis provide useful resources for future comparative botanical studies.

## 4. Materials and Methods

### 4.1. Chemicals and Reagents

Magnolol, chavicol, and other reagents were procured from Solarbio. Methanol and acetonitrile for HPLC were acquired from Sigma-Aldrich (St. Louis, MO, USA). The host bacteria *Escherichia coli* originated from Miaoling (Wuhan, China).

### 4.2. Preparation of Transcriptome Materials

*M. officinalis* plant samples were procured from the Hubei Academy of Agricultural Sciences located in Enshi, Hubei Province, China (Postcode: 445000). Due to the widespread reports [55,56,57] suggesting that 2–5-year-old *M. officinalis* contain negligible or extremely low levels of magnolol, and it has been determined that plants older than 16 years are preferable for the extraction of magnolol. Furthermore, the concentration of magnolol varies among different parts of the plant [24,58], with approximate contents of 1–5% in the roots, around 1% in the bark, and 0.1–0.5% in the leaves. Thus, we collected samples from both 2–5-year and 16-year-old plants, including the bark, leaves, and roots, with 3–4 replicates each for the detail analysis, as outlined in our sampling chart (Table S1).

Total RNA extraction followed standard protocols using TRIzol^®^ Reagent (Life Technologies, Carlsbad, CA, USA) and further purification with the RNeasy kit (Qiagen, Hilden, Germany). Subsequent treatment with RNase-free DNase I (Invitrogen, Pittsburgh, PA, USA) ensured the removal of residual DNA. The purity and quality of the RNA were determined by measuring the OD260/230 ratio using a NanoDrop 2000 (Thermo Fisher, Waltham, MA, USA), while RNA integrity was determined using an Agilent 2100 Bioanalyzer (Agilent Technologies, Santa Clara, CA, USA) based on the RNA Integrity Number (RIN). Only RNAs with RIN values ≥ 8.5 were used for library generation.

### 4.3. Transcriptome Analysis

Sequencing libraries were individually constructed and sequenced for each sample on the Illumina platform, yielding 279.3 Gb of raw paired-end sequence reads. HISAT2 (version 2.0.4) aligned RNA-seq data to the reference genome [59], with subsequent transcript assembly using StringTie (version 2.1.4) after preprocessing with fastp (version 0.20.1) to remove low-quality reads and splicing adapter sequences [60,61]. Genes with a read count below 3 and high intragroup variation (coefficient > 0.8) were excluded. The gene expression levels were quantified in TPM with StringTie v1.3.3 [62]. Differentially expressed genes (DEGs) between the tissues were identified through DESeq2 [63] (https://bioconductor.org/packages/release/bioc/html/DESeq2.html, accessed on 1 December 2023) with a threshold of *p* < 0.05 and a fold change >2.

### 4.4. Gene Cloning, Protein Expression, and Protein Purification

The genes, including *MoSKU5F*, *MoLAC7B*, *MoLAC4A*, *MoLAC4B*, *MoLAC17F*, and *MoLAC14*, tagged by 6 × histidine at the 3’-end, were synthesized by Wuhan Genecreate Biotechnology Co., Ltd. (Wuhan, China), cloned into the pET-28a(+) vector using the NdeI and XhoI restriction sites at the 5’- and 3’-ends, and transformed into *E. coli* BL21(DE3) for protein expression. The culture was incubated at 37 °C for 8 h in LB broth with 100 μg/mL kanamycin. Subsequently, isopropyl-β-D-thiogalactoside (IPTG) induction (0.5 mM) and the addition of copper (II) ions (Cu^2+^) (1 mM) preceded a 16 h incubation at 16 °C. Pelleted cells were then resuspended in a lysis buffer (50 mM Phosphate-buffered saline, pH 7.5) and subsequently disrupted using an ultra-high-pressure sterilizer at a pressure range of 700–1000 psi for 3 min at 4 °C. The supernatant was purified using Ni NTA affinity chromatography, eluted with 50 mM potassium phosphate solution containing 50 mM, 100 mM, 200 mM, and 300 mM imidazole, and further concentrated and replaced with 50 mM potassium phosphate solution (pH 7.5). The protein concentration was determined using a BCA protein concentration detection kit, and the purified protein was stored at −80 °C after rapid freezing in liquid nitrogen. Furthermore, SDS-PAGE gel verification confirmed the purity of the obtained protein.

### 4.5. Structural Simulation and Molecular Modification of MoLAC14

All structural models within this study were generated using the the Openfold platform (https://github.com/aqlaboratory/openfold, accessed on 11 December 2023). The chavicol structure was sourced from PubChem (CID:68148) and processed using AutoDockTools for processing protein receptors and small ligand structures. Substrate small molecules were docked with the MoLAC14 enzyme and its mutants through the Watvina platform (https://github.com/biocheming/watvina, accessed on 12 December 2023), by using a docking box built through a cube of 40 nm edge length.

Molecular dynamics simulations were conducted using the GROMACS 2020.6 package (https://github.com/bioexcel/gromacs-docker/releases/tag/2020.6-1, accessed on 12 December 2023), utilizing the Amber14sb forcefield for proteins and the GAFF forcefield (general amber force field) for small molecule substrates [64]. Post-docking, substrate–small molecule complexes were inserted into a cubic periodic water box with a minimum boundary distance of 10 Å, using the TIP3P model for water molecules. Sodium ions were used to balance system charges. Distant static electronic interactions were conducted using the particle mesh Ewald method with a boundary of 1.0 nm [65]. The potential energy minimum of the system was explored using a maximum of 5000 steps of energy minimization via the steepest descent method. This was followed by 100 ps of restraint dynamics stages, with the initial state of the dynamic simulation output. Finally, a 20 ns molecular dynamics simulation was carried out.

Trajectory analysis was performed on the stabilized 10–20 ns trajectory, examining rmsd to evaluate trajectory stability and residue fluctuation. Structural analysis was conducted on the largest cluster structure during 10–20 ns. PyMol (https://github.com/schrodinger/pymol-open-source, accessed on 12 December 2023) was employed for structural visualization and analysis. PositionScan (https://github.com/theone4ever/PCPS/blob/master/Vaaan.PictureCode.PositionScan.ObjecDetector/Properties/Resources.Designer.cs, accessed on 12 December 2023) from foldx and Cartesian_ddG from Rosetta (https://github.com/dohlee/snakemake-cartesian-ddg, accessed on 12 December 2023) were used for virtual saturation mutation of all MoLAC14 residues. Designs that emerged during computation through both methods were considered results for the current round of positive selection.

### 4.6. Enzyme Assay

The enzymatic catalysis of laccase was investigated under various conditions in a 200 μL reaction volume, containing the substrate chavicol, laccase enzyme, and divalent copper ions. Different parameters, including substrate concentration, (1–10 g/L), reaction times (6–36 h), temperatures (15–80 °C), and buffer solutions (citrate, phosphate, and Tris-HCl, pH 4.5–9), were tested to determine optimal conditions. Additionally, the effect of copper ion concentration on laccase-catalyzed paeonol production was evaluated. The 50 mM potassium phosphate solution required for the enzyme assay was filtered. Chavicol stock solutions were prepared in methanol and added to achieve the desired final concentrations in the reactions. The reaction mixtures were shaken at 1000 rpm for the specified time at the designated temperature.

After the enzymatic reaction, the addition of 200 μL of ethyl acetate terminated the reaction and extracted the products. Centrifugation at 12,000 rpm for 10 min separated the layers, and the upper ethyl acetate layer (100 μL) containing the products was dried under vacuum. The residue was reconstituted in 100 μL methanol for subsequent HPLC analysis.

The thermal stability of wild-type and mutant laccase enzymes was examined by heating enzyme solutions (2 mg/mL) at 60 °C for 24 h. After heating, 1 mM CuSO_4_ and 1 g/L chavicol dissolved in methanol were added to the sample and incubated at 60 °C for 3 h. Paeonol production was subsequently measured as described earlier. Higher paeonol yields indicated greater retained enzyme thermal stability.

For the chavicol catalysis reactions, crude enzyme extracts containing coexpressed APP1 and CAAT were prepared in PBS buffer. Acetyl coenzyme A, p-coumaryl alcohol, and NADPH were added to final concentrations of 0.5 mM. The reactions were performed at 30 °C for 5 h in a 50 μL system. After the reaction, 50 μL of ethyl acetate was added, shaken at 1000 rpm for 10 min, and the resultant products were extracted. The ethyl acetate was dried completely under vacuum and reconstituted in 50 μL methanol. Samples were centrifuged at 12,000 rpm and analyzed using HPLC. A C18 column was used with a water mobile phase and 80% methanol eluent. Detection was performed at 292 nm and 40 °C at a flow rate of 0.6 mL/min.

### 4.7. Analytical Methods

HPLC detection utilized a Waters 2998 (Sunnyvale, CA, USA) equipped with a 2695 PDA detector and a C18 column (4 mm × 250 mm). An 80% methanol solution was used as the mobile phase, with the injection volume 10 μL. The absorbance was monitored at 294 nm with a flow rate of 0.6 mL/min and a column temperature of 37 °C. The running time was 30 min. MS conditions on an Agilent 1200 HPLC system with a Bruker-MicrOTOF-II mass spectrometer included an ESI-positive ion source. The molecular weight was scanned within the range of 50–1000 (*m*/*z*), and the spray voltage was set at 4500 V. The capillary temperature, nitrogen flow rate, drying temperature, and atomization pressure were set to 400 °C, 6 mL/min, 180 °C, and 1 bar, respectively.

## Figures and Tables

**Table 1:** Kinetic parameters of the L532A mutant and MoLAC14.

Strains	Vmax	Km (mol/L)	Kcat (1/S)	Km/Kcat
L532A	8.76 × 10^−3^	8.76 × 10^−9^	15.33	5.71 × 10^−10^
MoLAC14	3.36 × 10^−3^	3.36 × 10^−10^	5.89	5.71 × 10^−11^

## Data Availability

The data presented in this study are available on request from the corresponding author. The data are not publicly available due to ethical restrictions.

## Supplementary Materials

The following supporting information can be downloaded at: https://www.mdpi.com/article/10.3390/molecules29030587/s1, Table S1: RNA sampling and raw data quality; Table S2: Gene annotation status; Table S3: Differential expression analysis; Table S4: Genes list from *Magnolia officinalis* derived from this work; Table S5: Plasmid collection derived from this study; Table S6: Strain inventory from this study; Table S7: Gene expression levels related to the conversion of tyrosine into chavicol; Figure S1: Principal component analysis between tissues and ages; Figure S2: GO enrichment analysis in biological processes; Figure S3: GO enrichment analysis in cellular components; Figure S4: GO enrichment analysis in molecular functions; Figure S5: Thirty potential laccase genes identified in *M. officinalis* by transcriptome analysis; Figure S6: Comparative analysis of the laccase gene family in *M. officinalis* versus *Arabidopsis thaliana* and other closely related species; Figure S7: MS analysis of the by-product. Its molecular formula was C_27_H_26_O_3_ (RT = 13.8 min), hypothesized to be the trimer Dunnianol or its isomer, derived from chavicol; Figure S8: The influence of heat treatment time on thermal stability and alanine scanning of residues within 5 Å of the substrate for MoLAC14; Figure S9: Genes with multiple copies involved in the synthesis from tyrosine to magnolol.
