# Supplementary material for: Identification and Validation of Magnolol Biosynthesis Genes in Magnolia officinalis
Source: Molecules. 2024 Jan 25;29(3):587. doi: 10.3390/molecules29030587 (PMC10856379; doi:10.3390/molecules29030587)
Supplement: Supplementary file 1 [file molecules-29-00587-s001.zip › molecules-2811965-supplementary.pdf]

Identification and validation of magnolol biosynthesis genes in *Magnolia officinalis*

Yue Yang[1,†], Zihe Li[1,†], Hang Zong[1], Shimeng Liu[2], Qiuhui Du[2], Hao Wu[2], Zhenzhu Li[1], Xiao Wang[2], Lihui Huang[2], Changlong Lai[2], Meide Zhang[3], Wen Wang [1,*], Xianqing Chen[2,*]

Additional files
Table S1 RNA Sampling and Raw Data Quality;
Table S2 Gene Annotation Status;
Table S3 Differential Expression Analysis;
Table S4 Genes list from *Magnolia officinalis* derived from this work;
Table S5 Plasmids collection derived from this study;
Table S6 Strains inventory from this study;
Table S7 Gene expression levels related to the conversion of tyrosine into chavicol;
Figure S1 Principal Component Analysis between tissues and ages;
Figure S2 GO Enrichment Analysis in Biological Processes;
Figure S3 GO Enrichment Analysis in Cellular Components;
Figure S4 GO Enrichment Analysis in Molecular Functions;
Figure S5 30 potential laccase genes identified in *M. officinalis* by transcriptome analysis;
Figure S6 Comparative analysis of the laccase gene family in *M. officinalis* versus *Arabidopsis thaliana* and other closely related species;
Figure S7 MS analysis of the by-product. Its Molecular formula was $C_{27}H_{26}O_3$ (RT= 13.8 min), hypothesized to be the trimer Dunnianol or its isomer, derived from chavicol;
Figure S8 The influence of heat treatment time on thermal stability and Alanine scanning of residues within 5 Å of the substrate for MoLAC14;
Figure S9 Genes with multiple copies involved in the synthesis from tyrosine to magnolol.

Table S1 RNA Sampling and Raw Data Quality

Sampling Tissue	Sample Name	total data (Gb)	mapping rate	Q30 reads rate
16-year-old tree leaves	l16_1	7.02	83.83%	97.65%
16-year-old tree leaves	l16_2	7.52	80.98%	97.92%
16-year-old tree leaves	l16_3	7.07	82.99%	97.70%
16-year-old tree leaves	l16_4	7.47	83.10%	97.84%
16-year-old tree bark	e16_1	8.11	86.16%	97.24%
16-year-old tree bark	e16_2	7.89	86.67%	97.28%
16-year-old tree bark	e16_3	5.08	86.34%	97.32%
16-year-old tree bark	e16_4	6.18	84.97%	97.22%
16-year-old tree roots	r16_1	7.24	86.14%	97.20%
16-year-old tree roots	r16_3	7.17	86.24%	97.17%
16-year-old tree roots	r16_4	6.67	85.69%	97.47%
2-5 year-old tree leaves	l2_1	6.88	79.65%	97.77%
2-5 year-old tree leaves	l2_2	6.88	83.89%	97.75%
2-5 year-old tree leaves	l2_3	7.41	83.57%	97.54%
2-5 year-old tree bark	e2_1	7.15	78.69%	97.73%
2-5 year-old tree bark	e2_2	8.49	78.60%	98.04%
2-5 year-old tree bark	e2_3	8.63	79.67%	97.97%
2-5 year-old tree roots	r2_1	8.82	77.43%	97.86%
2-5 year-old tree roots	r2_2	9	86.14%	97.98%
2-5 year-old tree roots	r2_3	7.09	85.25%	98.02%

Table S2 Gene Annotation Status

Gene Annotation Status	Number
Number of Genes	19946
Number of mRNA	52692
Average Length of CDS	1224bp
Complete BUSCOs % of proteins	79.30%
Complete BUSCOs % of genome	86.20%

Table S3 Differential Expression Analysis

Analysis Groups for Differential Comparisons	Up/Down Regulation	Number of Differentially Expressed Genes
e16 vs e2	down	1563
e16 vs e2	up	682
e16 vs l16	down	4685
e16 vs l16	up	3698
e16 vs r16	down	394
e16 vs r16	up	356
e2 vs l2	down	3394
e2 vs l2	up	3373
e2 vs r2	down	2280
e2 vs r2	up	1264
l16 vs l2	down	191
l16 vs l2	up	587
r16 vs l16	down	4139
r16 vs l16	up	3428
r16 vs r2	down	1573
r16 vs r2	up	371
r2 vs l2	down	3157
r2 vs l2	up	4439

Table S4. Genes list from *Magnolia officinalis* derived from this work

Gene name	Amino acid sequence
MoSKU5F	MTPTVNAKILRYKWDVKYQFKAPDCYRKLVIAINGKTPGPTILAQQ<br>GDTIIVELKNSLLTENVAIHWHGIRQIGTPWSDGTEGVSQCPITPGDTF<br>VYKFVVDRPGTYLYHAHYGMQREAGLYGSIRVSVPEGKTEPFTYDY<br>DRSIILTDWWHKSTYEQATGLSSLPFQWVNEPQSLLIQGKGRFDCSLV<br>TPSSSNISEICNASDPECGHYVLTVVPGKTYRLRIASLTSLSALNFEIEG<br>HNMTVVEADGHYVEPFIIKDLHIYSGETYSVLVKADQDPSRNYWATT<br>NVVSREPKTPTGLAIFNYYPNHPRKLPTTTPPTGPLWNDTNVRLAQS<br>LKIKSHKGYIHQPPLTSDRVMILLNTQNRINGFVRWSINNVSFTLPHTP<br>YLIALKENLTNVFDQTPPPETYDYANYNIYSTPNNTNASSTNSIYRLQ<br>FNSTVDIILQNANMMDPNKSETHPWHLHGHDFWVLGYGDGVFDPV<br>NDPKKYNLVDPIMKNTVPVHPYGWTALRFQADNPGVWAFHCHIEA<br>HYYLGMGVVFEEGVEKVGKLPSSIMGCGETKGFKRP
MoLAC7B	MIFFFFFLNFIHFQVGNLTVKPLCEERVVVAVNGQLPGPTISVREGDTL<br>VVHVINESPYNVTIHWHGIFQQLTGWADGPAYVTQCPILPGDSYTYR<br>FNIIKQQGTLWWHAHVSWLRATVYGALIIHPRQHRPYPFIKPHKQFPI<br>ILGEWWNANVVDVENEARLNGVAPNISDAYTINGRPGDLYPCANNN<br>TYKIQVAHGKTYLLRIINAALNNELFFKIAGHNLTVVGIDALYTDPYT<br>TDVVVLAPGQTTGVLLVANATPGSYYMAAHPYATAPIPFDNTTTTAIL<br>QYKGATSVAAPLMPVLPQFNDTPTAHKFYSNITALSRRGSEVPLHVD<br>EHMFITVGLGLEPCGQNATCTRPAASMNNASFQLPTTLSMLQAHFSG<br>VKGIYTDDFPDTPPLVFDYTNANASQLVSTTLKSTRVKRLAYNTTVE<br>MVFQNTAILSVENHPIHLHGFNFFVVAQGFGNYDNTTHRNMFNLVN<br>PQRRNTIAVPVGGWAVVRFQANNPGVWLMHCHLDVHLTWGLATAF<br>VVDNGPTISSTLPPPPPDLPRC
MoLAC4A	MKNTTRLCQSKPIVTVNGQFPGPTLYAREGDNVLVKVVNHVKYNVT<br>IHWHGIRQFRTGWADGPAYITQCPIQPRNQYIYNFTITGQRGTLWWH<br>AHILWLRATVHGAFVILPNLPAPYVPYPFPTPDKEVVVILGEWWKSDI<br>EVIDESLNSGVSPNVSDAHTINGHPGPFSNCSSNGGFTLKVRRGKTY<br>MLRVINAALNDELFFKVAGHKLTVVEVDATYTKPFKTDTLLITPGQT<br>TNVLLTADRVPGRYLVVVSPFKDSPIPVDNTTGTAILHYSGMLGALPT<br>TLTTPPPQNATPVATNFNAALRSLNSKKFPANVPLTVDHSLLFTVGVG<br>FIPCSTCNNGKRLVVFTSDFPGNPPITFNYTGTLPSNPRTTNGTRLYRL<br>AYNSTVQIVFQDTTFISPENHPIHLHGFNFFAVGRGLGNFNPKTDTAN<br>FNLVDPVERNTIGVPSGGWTAIRFRADNPGVWFLHCHLEVHTTWGL<br>KMAFVVDNGKGPNQSLLPPPSDLPAC
MoLAC4B	MELWVRALVLVACLLLPTVVECRVRHYKFNVVMKNTTRLCQSKPIV<br>TVNGQFPGPTLYAREGDNVVVKVVNHVKYNVTVHWHGIRQLRTG<br>WADGPAYITQCPIQPGNQYIYNFTITGQRGTLWWHAHILWLRATVHG

	AIVILPKLRVPYVPCEWWKSDTEAVINEALNSGLAPNVSDAHTINGH<br>PGPFSNCSSQGGFTLKVRRGKTYMLRLINAALNDELFFKVAGHKLTV<br>VEVDAAYTKPFKTDTVLIAPGQTTNVLLTTDRAPGRYLVAVSPFMDS<br>PIAVDNTTGTAILHYSGMLGASPTTLTTPPPQNATPIATNFNAALRSLN<br>SKKFPANVPLTVDHSLLFTVGLGVSPCSTCKNGIRVAADINNVSFVMP<br>TTALLQAHYFNISGVFTNDSPAIRRSPSITPVQCLRTCKPQMGLGFIG<br>WRTIQRCRLSCKIPALYRQKTIRSTFTVSISSPSVGGGEFQSKERYR
MoLAC17F	MATVNGRYPGPKIMAREGDRVVVEVANNVQNNVTIHWHGIRQLQS<br>GWADGPAYITQCPIQTGQTYVYNFTITGQRGTLFWHAHFSWMRATL<br>YGPLIVLPMQNASYPFGQPDEEVPIIFGEWWNANTEAVINQSLQTGA<br>GPNVSDAYTINGKPGPLYNCSAKDTFKLKVKPGKTYLLRLINAALND<br>ELFFSIANHTITVVEVDAIYIKPFNTKTILIAPGQTTNVLLNTKPHYPN<br>ATFFMSARPYLTGLGTFDNTTTVGLLEYEKPNSSLPSTNLPIFKPSLPA<br>LNDTSYAANYSQRLRSLANSQFPANVPLTVDKRFFFTIGLGADPCPK<br>NQTCQGPNGTKFSASINNISFVLPTTALLQAHFFGQSNGVYNTSFPNN<br>PPLPHGTKVVVLPFNTTVELVLQDTSILGVESHPLHLHGFNFFVVGQ<br>GFGNFDRKKDPAKFNLVDPIERNTVGVPSGGWVVLRFRADNPGVWF<br>LHCHFDVHLSWGLKMAWVVLDGKLPNQKLLPPPSDLPKC
MoLAC14	MATVNGRYPGPKIMAREGDRVVVEVANNVQNNVTIHWHGIRQLQS<br>GWADGPAYITQCPIQTGQTYVYNFTITGQRGTLFWHAHFSWMRATL<br>YGPLIVLPMQNASYPFGQPDEEVPIIFGEWWNANTEAVINQSLQTGA<br>GPNVSDAYTINGKPGPLYNCSAKDTFKLKVKPGKTYLLRLINAALND<br>ELFFSIANHTITVVEVDAIYIKPFNTKTILIAPGQTTNVLLNTKPHYPN<br>ATFFMSARPYLTGLGTFDNTTTVGLLEYEKPNSSLPSTNLPIFKPSLPA<br>LNDTSYAANYSQRLRSLANSQFPANVPLTVDKRFFFTIGLGADPCPK<br>NQTCQGPNGTKFSASINNISFVLPTTALLQAHFFGQSNGVYNTSFPNN<br>PPLPHGTKVVVLPFNTTVELVLQDTSILGVESHPLHLHGFNFFVVGQ<br>GFGNFDRKKDPAKFNLVDPIERNTVGVPSGGWVVLRFRADNPGVWF<br>LHCHFDVHLSWGLKMAWVVLDGKLPNQKLLPPPSDLPKCLEHHHH<br>HHEF

Table S5 Plasmids collection derived from this study

Plasmids	Genotype
pET28a	pBR322 ori with pT7; KanR
pET28a- MoSKU5F	pET28a vector, NdeI- MoSKU5F -XhoI
pET28a-MoLAC7B	pET28a vector, NdeI- MoLAC7B -XhoI
pET28a - MoLAC4A	pET28a vector, NdeI- MoLAC4A -XhoI
pET28a- MoLAC4B	pET28a vector, NdeI- MoLAC4B -XhoI
pET28a -MoLAC17F	pET28a vector, NdeI- MoLAC17F -XhoI
pET28a -MoLAC14	pET28a vector, NdeI- MoLAC14 -XhoI

Table S6 Strains inventory from this study

Strains	Genotype
BL21(DE3)	F-; ompT; hsdS（rBB-mB－);gal;dcm（DE3）
S5F	BL21(DE3) carrying pET28a- MoSKU5F
S7B	BL21(DE3) carrying pET28a-MoLAC7B
S4A	BL21(DE3) carrying pET28a - MoLAC4A
S4B	BL21(DE3) carrying pET28a- MoLAC4B
S17F	BL21(DE3) carrying pET28a -MoLAC17F
S14	BL21(DE3) carrying pET28a -MoLAC14

Table S7 Gene expression levels related to the conversion of tyrosine into chavicol

Gene name	Adjust p value	Log2(Fold change)	Group	Type
Mo4CL1	0.00011	-2.65164	e2 vs l2	DOWN
Mo4CL1	0.00038	-2.63430	e2 vs r2	DOWN
Mo4CL1	0.03563	-1.83893	e16 vs r16	DOWN
Mo4CL1	0.00003	-2.41695	e16 vs l16	DOWN
Mo4CL2	0.00153	3.05158	r16 vs l16	UP
Mo4CL2	0.00198	-3.73052	e16 vs r16	DOWN
Mo4CL2	0.00002	4.31002	r2 vs l2	UP
Mo4CL2	0.00009	-4.32183	e2 vs r2	DOWN
Mo4CL3	0.01355	-1.01245	e2 vs r2	DOWN
Mo4CL3	0.00001	-1.57914	e2 vs l2	DOWN
Mo4CL3	0.00004	-1.25750	e16 vs l16	DOWN
MoADH1	0.00000	5.25155	r2 vs l2	UP
MoADH1	0.00000	6.57484	r16 vs l16	UP
MoADH1	0.00000	7.54652	e16 vs l16	UP
MoADH1	0.00000	6.12294	e2 vs l2	UP
MoADH2	0.00018	5.68845	e2 vs l2	UP
MoADH2	0.00609	4.22823	r2 vs l2	UP
MoADH2	0.00001	5.23369	e16 vs l16	UP
MoADH5	0.00018	-8.17421	e2 vs l2	DOWN
MoADH5	0.00471	-6.84306	e2 vs r2	DOWN
MoADH5	0.00754	-6.94765	r16 vs r2	DOWN
MoADH5	0.00005	-8.11174	r16 vs l16	DOWN
MoADH5	0.00000	-12.68389	e16 vs l16	DOWN
MoADH6	0.00717	1.45115	r16 vs l16	UP
MoADH6	0.01044	1.49143	r2 vs l2	UP
MoADH7	0.00195	-1.46646	e16 vs r16	DOWN
MoADH7	0.00336	1.27271	e16 vs e2	UP
MoADH7	0.00000	6.70898	r16 vs l16	UP
MoADH7	0.00000	5.24252	e16 vs l16	UP
MoADH7	0.00000	-2.12274	e2 vs r2	DOWN
MoADH7	0.00000	3.81390	e2 vs l2	UP
MoADH7	0.00000	5.93664	r2 vs l2	UP
MoADH8	0.02773	-2.18813	r16 vs r2	DOWN
MoADH8	0.00000	-5.29536	r16 vs l16	DOWN
MoADH8	0.00000	-4.82515	e2 vs l2	DOWN
MoADH8	0.00001	-3.49974	r2 vs l2	DOWN
MoADH8	0.00000	-4.49300	e16 vs l16	DOWN
MoAPS2	0.00003	1.85022	r2 vs l2	UP
MoAPS2	0.00000	3.24434	r16 vs l16	UP
MoAPS2	0.00000	2.57820	e2 vs l2	UP
MoAPS2	0.00000	3.91400	e16 vs l16	UP

MoAPS3	0.00037	1.43570	e2 vs l2	UP
MoAPS3	0.00217	1.23928	r2 vs l2	UP
MoCAAT1A	0.00088	-3.71703	r16 vs l16	DOWN
MoCAAT1A	0.00220	-4.24963	r16 vs r2	DOWN
MoCAAT1A	0.00537	-3.68754	e2 vs r2	DOWN
MoCAAT1A	0.00000	-5.14052	e16 vs l16	DOWN
MoCAAT1B	0.00441	-7.55774	e2 vs r2	DOWN
MoCAAT1B	0.02631	5.06656	r16 vs l16	UP
MoCAAT1B	0.00000	26.31881	r2 vs l2	UP
MoCAAT1B	0.00000	21.98887	l16 vs l2	UP
MoCAAT1B	0.00000	18.76107	e2 vs l2	UP
MoCAAT1C	0.00023	6.82140	e2 vs l2	UP
MoCAAT1C	0.00360	4.95372	r16 vs l16	UP
MoCAAT1C	0.01070	-4.15200	r16 vs r2	DOWN
MoCAAT1C	0.02583	-3.67866	e16 vs e2	DOWN
MoCAAT1C	0.00000	8.97930	r2 vs l2	UP
MoCAAT2A	0.00038	7.96667	r16 vs l16	UP
MoCAAT2A	0.00047	8.60809	r2 vs l2	UP
MoCAAT2A	0.03552	4.63246	e16 vs l16	UP
MoCAAT2B	0.00034	-4.72147	r16 vs r2	DOWN
MoCAAT2B	0.00061	3.97621	r16 vs l16	UP
MoCAAT2B	0.01377	-3.76108	e16 vs r16	DOWN
MoCAAT2B	0.04394	-2.72911	e2 vs r2	DOWN
MoCAAT2B	0.00000	8.43717	e2 vs l2	UP
MoCAAT2B	0.00000	-5.75344	e16 vs e2	DOWN
MoCAAT2B	0.00000	11.16628	r2 vs l2	UP
MoCCR1	0.00983	1.11009	e16 vs l16	UP
MoCCR2	0.00000	2.53627	e16 vs l16	UP
MoCCR2	0.00000	2.56286	e2 vs l2	UP
MoCCR2	0.00000	1.98188	e16 vs r16	UP
MoCCR2	0.00000	2.08770	e2 vs r2	UP
MoCCR4	0.00073	-2.76387	e16 vs l16	DOWN
MoCCR4	0.00253	-3.37222	r16 vs r2	DOWN
MoCCR4	0.01955	-2.55152	e2 vs r2	DOWN
MoCCR4	0.02436	-2.08095	r16 vs l16	DOWN
MoCCR5	0.00056	-2.65549	r2 vs l2	DOWN
MoCCR5	0.00711	-2.33089	e2 vs r2	DOWN
MoCCR5	0.00000	-6.08362	r16 vs l16	DOWN
MoCCR5	0.00002	-3.49136	e16 vs e2	DOWN
MoCCR5	0.00000	-4.98637	e2 vs l2	DOWN
MoCCR5	0.00000	-4.16135	r16 vs r2	DOWN
MoCCR5	0.00000	-7.74451	e16 vs l16	DOWN
MoCCR7	0.00020	-1.73743	e2 vs l2	DOWN
MoCCR7	0.00213	-1.34297	r16 vs l16	DOWN

MoCCR7	0.00005	-1.58691	e16 vs l16	DOWN
MoLAC11	0.00225	9.28048	e2 vs l2	UP
MoLAC11	0.00462	8.57096	r2 vs l2	UP
MoLAC11B	0.00312	10.68263	r2 vs l2	UP
MoLAC11C	0.00834	-7.08054	r16 vs r2	DOWN
MoLAC11C	0.01036	-6.46246	e2 vs r2	DOWN
MoLAC11C	0.00005	9.40439	r2 vs l2	UP
MoLAC12	0.00012	4.72317	r2 vs l2	UP
MoLAC12	0.00112	-4.41414	e2 vs r2	DOWN
MoLAC12	0.03048	-2.36973	e16 vs l16	DOWN
MoLAC12	0.03447	-3.36139	e16 vs r16	DOWN
MoLAC14	0.01384	-1.34620	e16 vs e2	DOWN
MoLAC14	0.02908	-1.24033	e2 vs r2	DOWN
MoLAC14	0.00000	-3.09393	e16 vs r16	DOWN
MoLAC14	0.00000	-4.80067	e2 vs l2	DOWN
MoLAC14	0.00000	-3.56035	r2 vs l2	DOWN
MoLAC14	0.00000	-5.81968	e16 vs l16	DOWN
MoLAC14	0.00000	-2.72575	r16 vs l16	DOWN
MoLAC17A	0.00391	-7.23990	r16 vs l16	DOWN
MoLAC17A	0.00000	21.09820	e2 vs l2	UP
MoLAC17A	0.00000	21.12668	r2 vs l2	UP
MoLAC17A	0.00000	26.19128	l16 vs l2	UP
MoLAC17B	0.00393	9.06086	r2 vs l2	UP
MoLAC17B	0.00755	-9.28498	e2 vs r2	DOWN
MoLAC17C	0.00203	8.70164	r2 vs l2	UP
MoLAC17C	0.00819	-8.12403	e2 vs r2	DOWN
MoLAC17D	0.00182	-9.52888	e2 vs r2	DOWN
MoLAC17D	0.01104	-5.66155	e16 vs l16	DOWN
MoLAC17D	0.01149	-6.27945	r16 vs l16	DOWN
MoLAC17D	0.03520	-6.82126	r16 vs r2	DOWN
MoLAC17E	0.00295	7.78954	r2 vs l2	UP
MoLAC17E	0.00917	-5.86836	e16 vs l16	DOWN
MoLAC17E	0.01159	-7.20640	e2 vs r2	DOWN
MoLAC17F	0.00000	20.69944	e2 vs l2	UP
MoLAC17F	0.00000	26.38554	r2 vs l2	UP
MoLAC17F	0.00000	21.67144	l16 vs l2	UP
MoLAC17G	0.00000	25.49176	r2 vs l2	UP
MoLAC17G	0.00000	25.06927	l16 vs l2	UP
MoLAC17G	0.00000	22.00275	e2 vs l2	UP
MoLAC17H	0.00000	-24.85875	e2 vs r2	DOWN
MoLAC17H	0.00000	22.80547	l16 vs l2	UP
MoLAC17H	0.00000	24.26372	r2 vs l2	UP
MoLAC17H	0.00000	19.80459	e16 vs e2	UP
MoLAC4A	0.01059	6.69037	e2 vs l2	UP

MoLAC4A	0.04884	-3.78672	e16 vs l16	DOWN
MoLAC4A	0.00001	10.89536	r2 vs l2	UP
MoLAC4A	0.00000	12.67617	l16 vs l2	UP
MoLAC4B	0.00532	7.56786	l16 vs l2	UP
MoLAC4B	0.00845	6.13606	r2 vs l2	UP
MoLAC4B	0.01127	-4.86184	e16 vs l16	DOWN
MoLAC4B	0.02636	-5.74269	e2 vs r2	DOWN
MoLAC4D	0.01809	-5.02734	e16 vs l16	DOWN
MoLAC4D	0.01810	7.40909	l16 vs l2	UP
MoLAC4D	0.02202	5.94669	r2 vs l2	UP
MoLAC4D	0.02852	-6.35589	e2 vs r2	DOWN
MoLAC5	0.00758	4.32074	e2 vs l2	UP
MoLAC5	0.00001	6.95588	r2 vs l2	UP
MoLAC7B	0.00032	-5.51454	e16 vs e2	DOWN
MoLAC7B	0.00502	-3.58912	e16 vs l16	DOWN
MoLAC7B	0.00558	-4.90892	e16 vs r16	DOWN
MoLAC7B	0.01362	3.76634	e2 vs l2	UP
MoLAC7B	0.00004	5.94557	r2 vs l2	UP
MoLAC7C	0.00250	-4.88321	e16 vs l16	DOWN
MoSKU5A	0.00014	-7.12740	e2 vs l2	DOWN
MoSKU5A	0.00200	-5.26393	r16 vs l16	DOWN
MoSKU5A	0.00888	-4.80831	r2 vs l2	DOWN
MoSKU5A	0.00004	-6.49165	e16 vs l16	DOWN
MoSKU5B	0.00106	-6.30297	e2 vs l2	DOWN
MoSKU5B	0.00159	-5.63847	r16 vs l16	DOWN
MoSKU5B	0.02842	-4.25886	r2 vs l2	DOWN
MoSKU5B	0.00002	-7.05039	e16 vs l16	DOWN
MoSKU5C	0.00016	4.24563	r16 vs l16	UP
MoSKU5C	0.00049	3.62396	e16 vs l16	UP
MoSKU5C	0.00000	5.71895	e2 vs l2	UP
MoSKU5C	0.00001	5.41397	r2 vs l2	UP
MoSKU5D	0.00046	3.98785	e2 vs l2	UP
MoSKU5D	0.00196	-3.69959	e2 vs r2	DOWN
MoSKU5D	0.00000	7.68744	r2 vs l2	UP
MoSKU5D	0.00006	4.03867	r16 vs l16	UP
MoSKU5E	0.00239	-19.08120	e16 vs e2	DOWN
MoSKU5E	0.00000	34.93341	r2 vs l2	UP
MoSKU5E	0.00000	-35.27709	r16 vs r2	DOWN
MoSKU5E	0.00000	26.81877	e2 vs l2	UP
MoSKU5G	0.02025	-3.17174	e2 vs r2	DOWN
MoSKU5G	0.00005	4.80840	r2 vs l2	UP
MoTAL1	0.00040	4.21362	r2 vs l2	UP
MoTAL1	0.04494	2.74670	e16 vs e2	UP
MoTAL1	0.00003	-5.32829	e2 vs r2	DOWN

MoTAL2	0.00148	-2.64807	e2 vs l2	DOWN
MoTAL2	0.03563	-2.20624	e16 vs r16	DOWN
MoTAL2	0.00006	-3.51313	e2 vs r2	DOWN
MoTAL3	0.00017	-3.09403	e16 vs l16	DOWN
MoTAL3	0.00315	2.89164	r2 vs l2	UP
MoTAL3	0.01762	-2.80856	r16 vs r2	DOWN
MoTAL3	0.03310	-2.34263	e16 vs e2	DOWN
MoTAL3	0.04635	-1.87878	r16 vs l16	DOWN
MoTAL4	0.00016	-4.85749	e2 vs l2	DOWN
MoTAL4	0.01492	-3.70720	e16 vs e2	DOWN
MoTAL4	0.00000	-7.54435	r16 vs l16	DOWN
MoTAL4	0.00000	-9.00244	e16 vs l16	DOWN
MoTAL4	0.00000	-7.38992	r2 vs l2	DOWN
MoTAL6	0.00743	-8.20077	e2 vs r2	DOWN
MoTAL6	0.02902	-7.56615	e16 vs r16	DOWN
MoTAL6	0.04355	6.47068	e2 vs l2	UP
MoTAL6	0.00001	11.51313	r16 vs l16	UP
MoTAL6	0.00000	14.67145	r2 vs l2	UP
MoTAL7	0.00088	-3.32183	e16 vs l16	DOWN
MoTAL7	0.00425	3.39259	r2 vs l2	UP
MoTAL7	0.03127	3.21731	l16 vs l2	UP

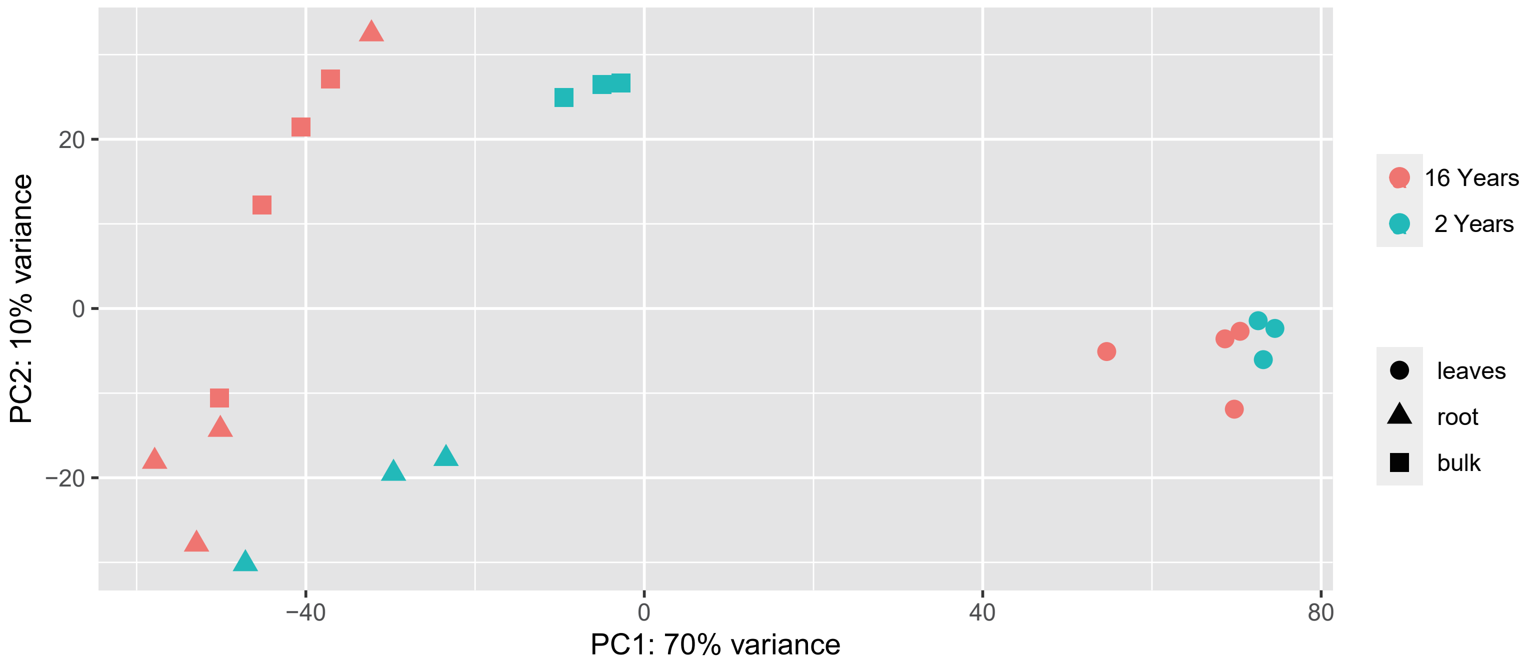

Figure S1 Principal Component Analysis between tissues and ages.

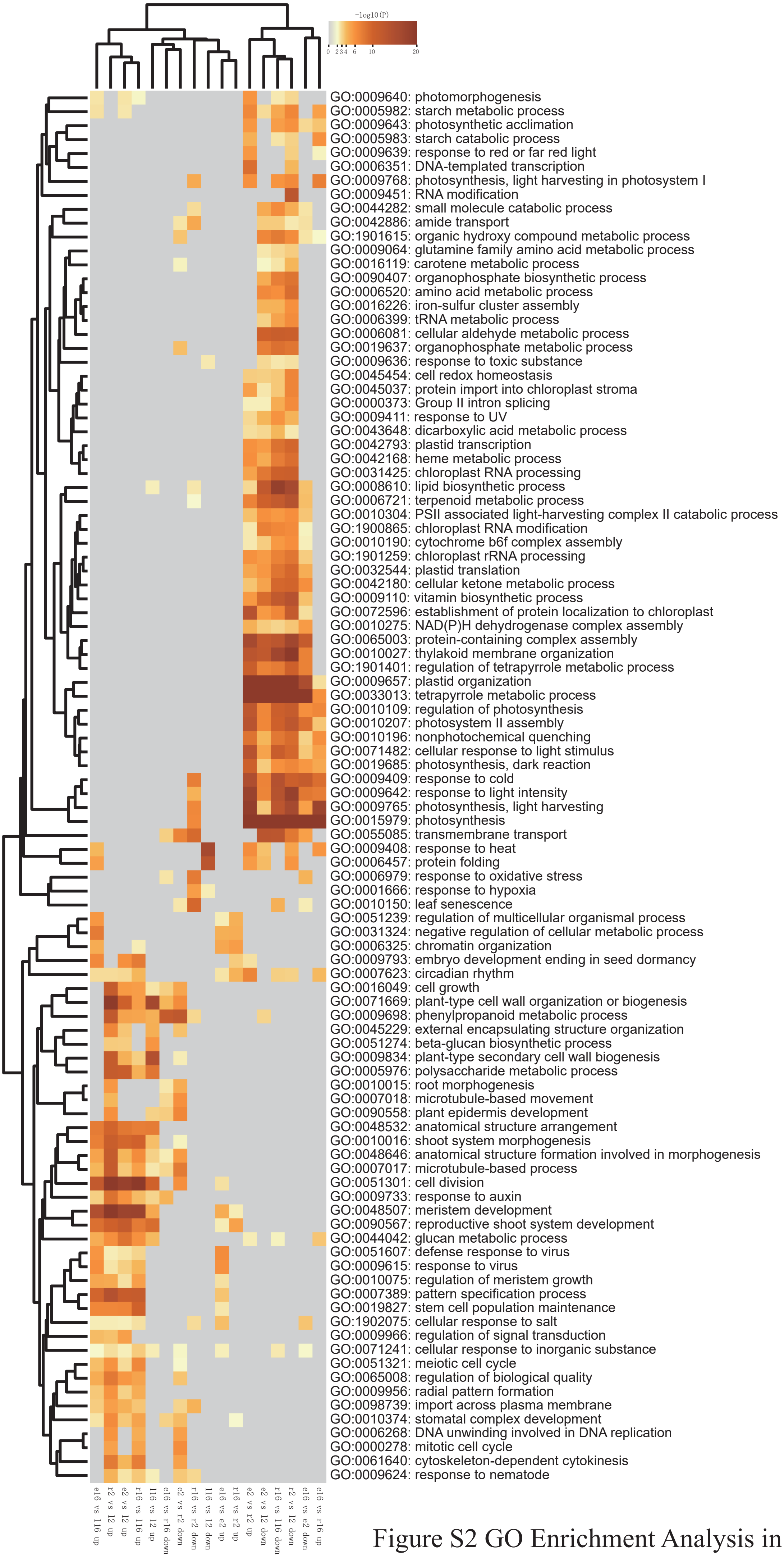

Figure S2 GO Enrichment Analysis in Biological Processes.

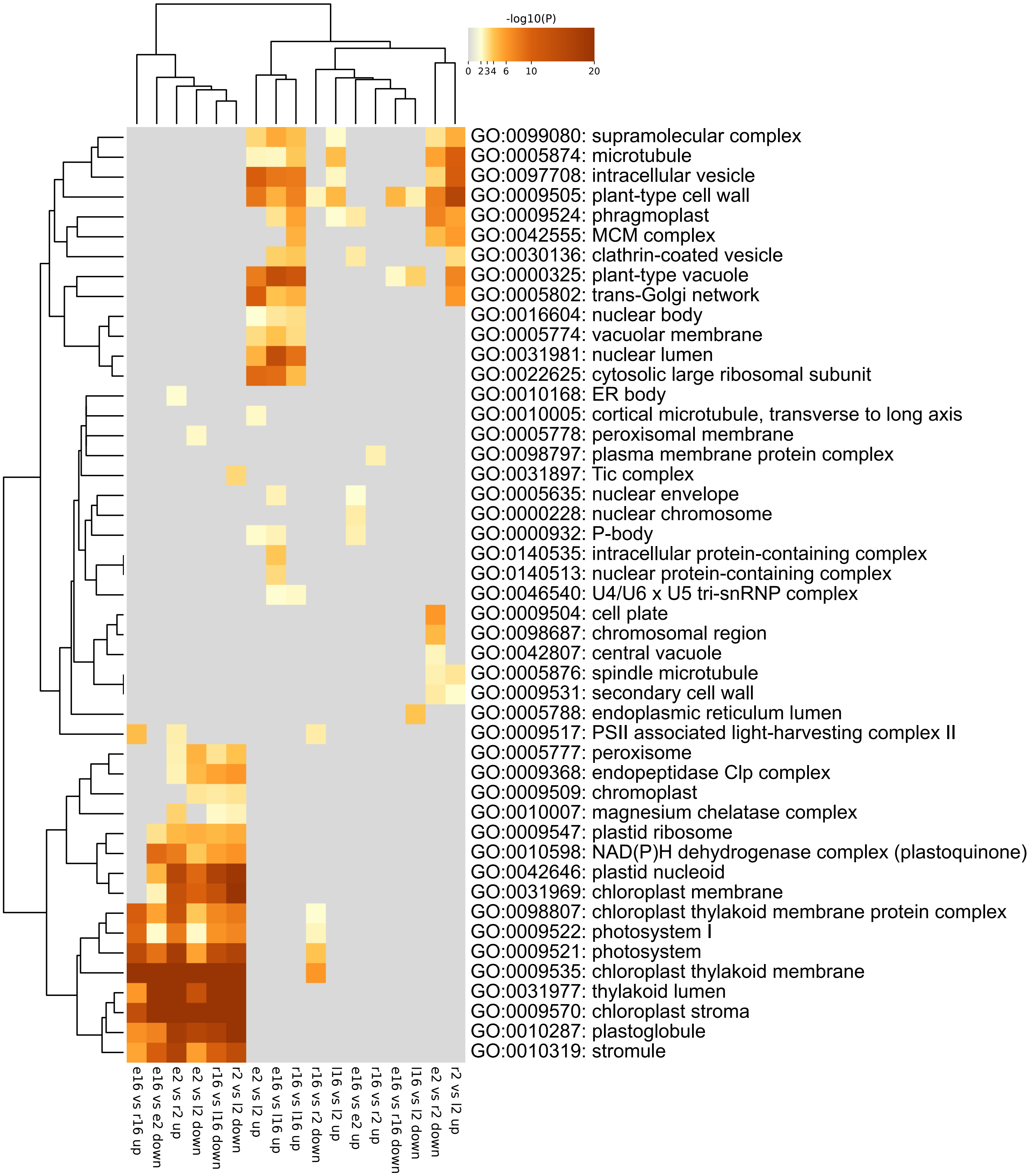

Figure S3 GO Enrichment Analysis in Cellular Components.

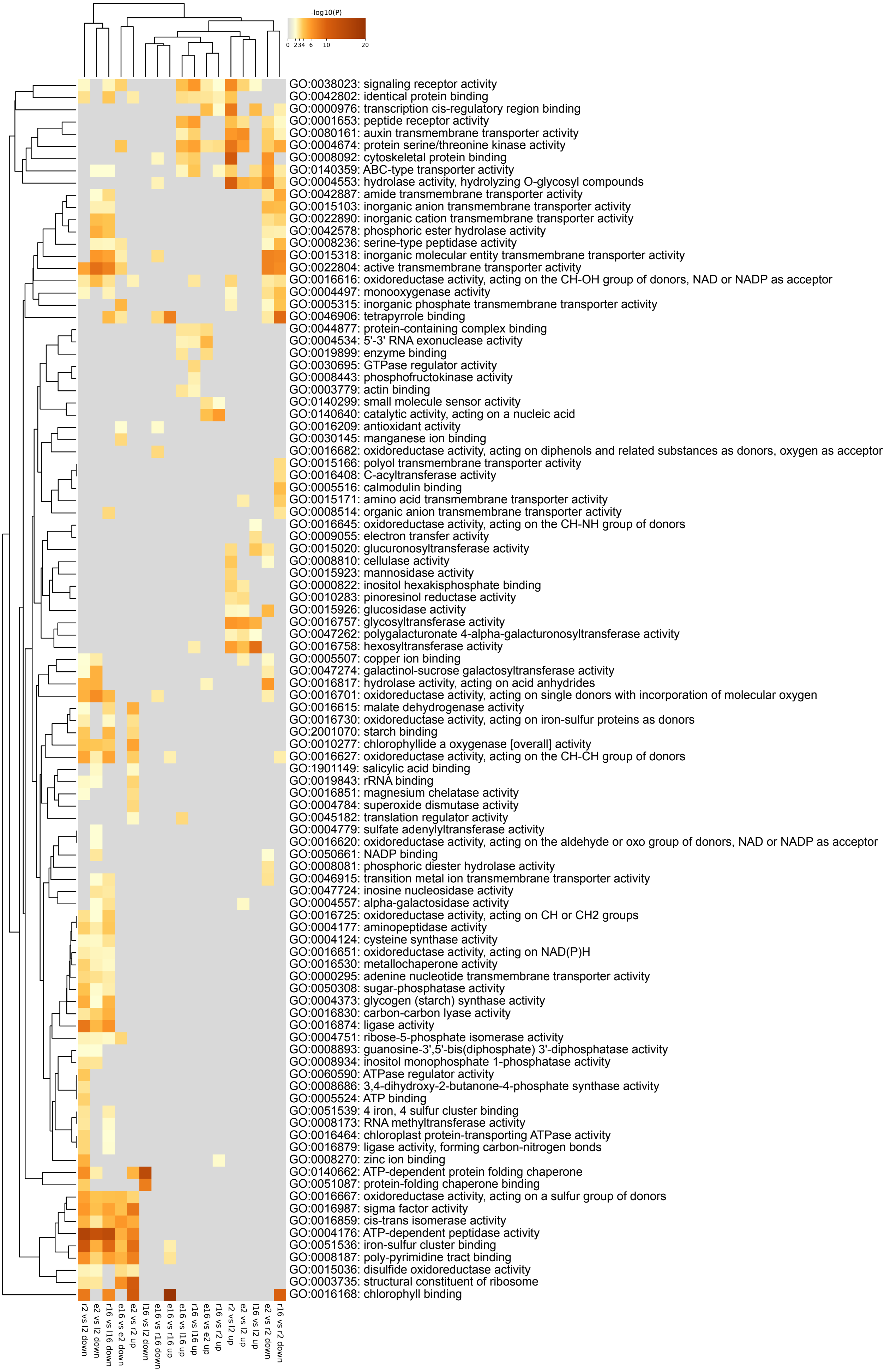

Figure S4 GO Enrichment Analysis in Molecular Functions.

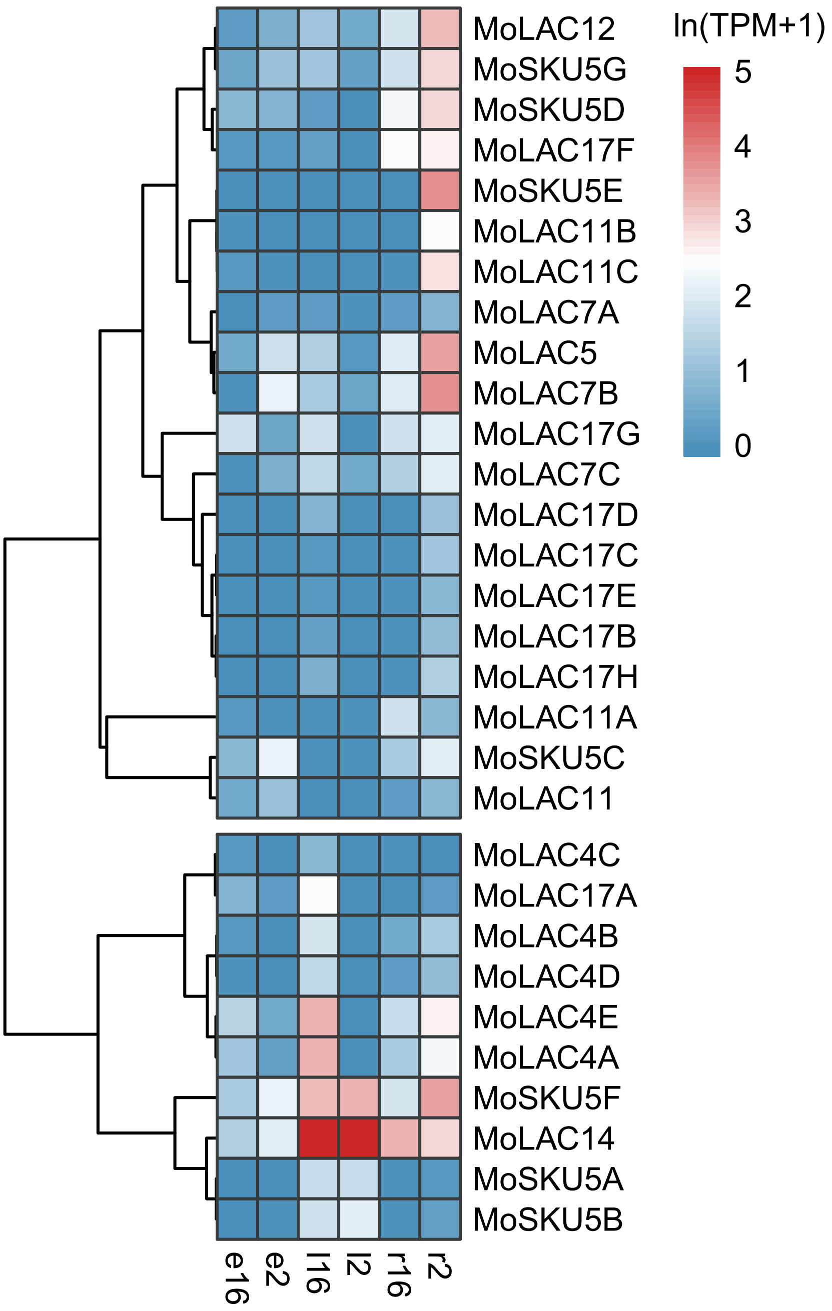

Figure S5 30 potential laccase genes identified in *M. officinalis* by transcriptome analysis.

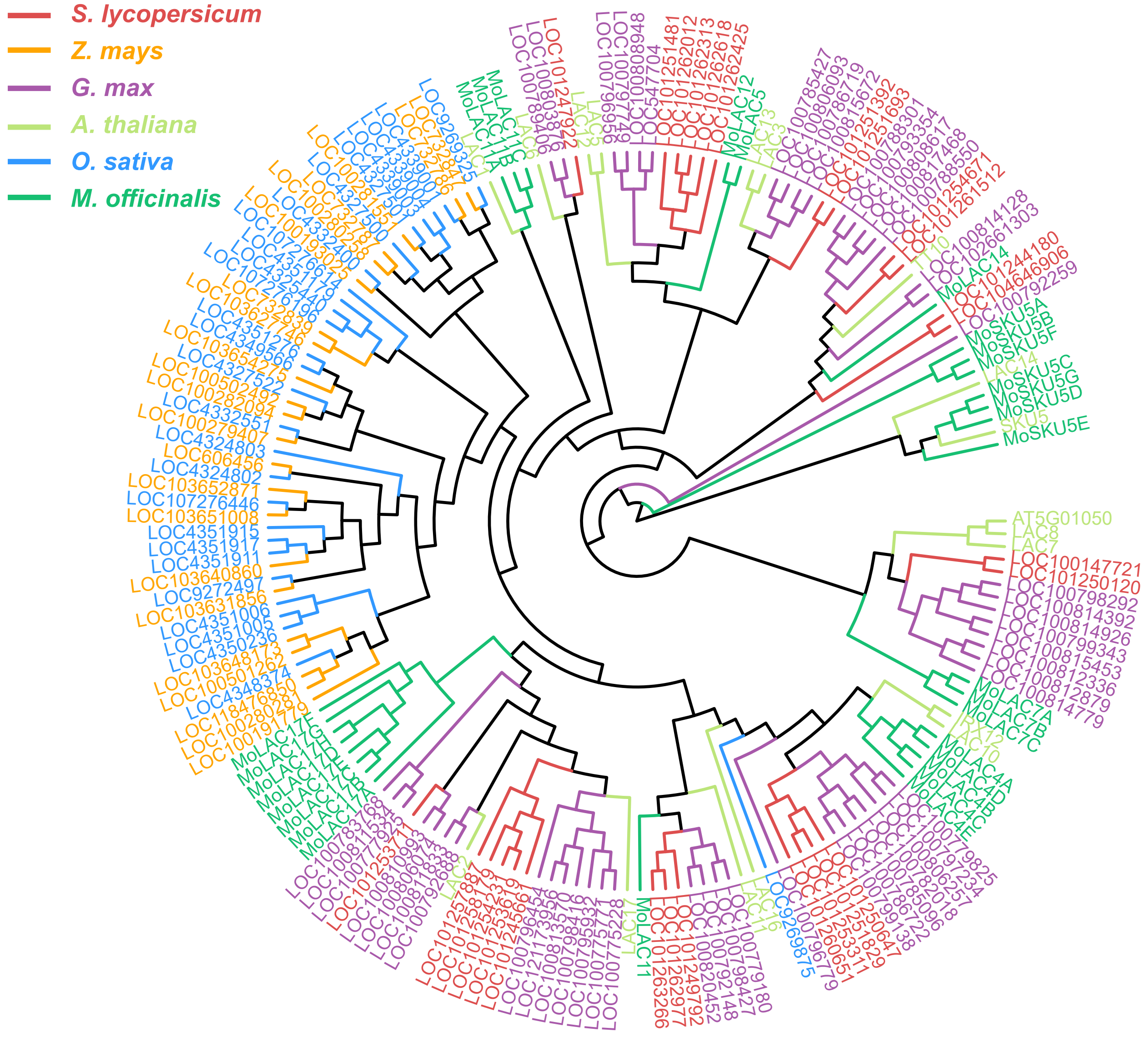

Figure S6 Comparative analysis of the laccase gene family in *M. officinalis* versus *Arabidopsis thaliana* and other closely related species.

$C_{27}H_{26}O_3$ -H

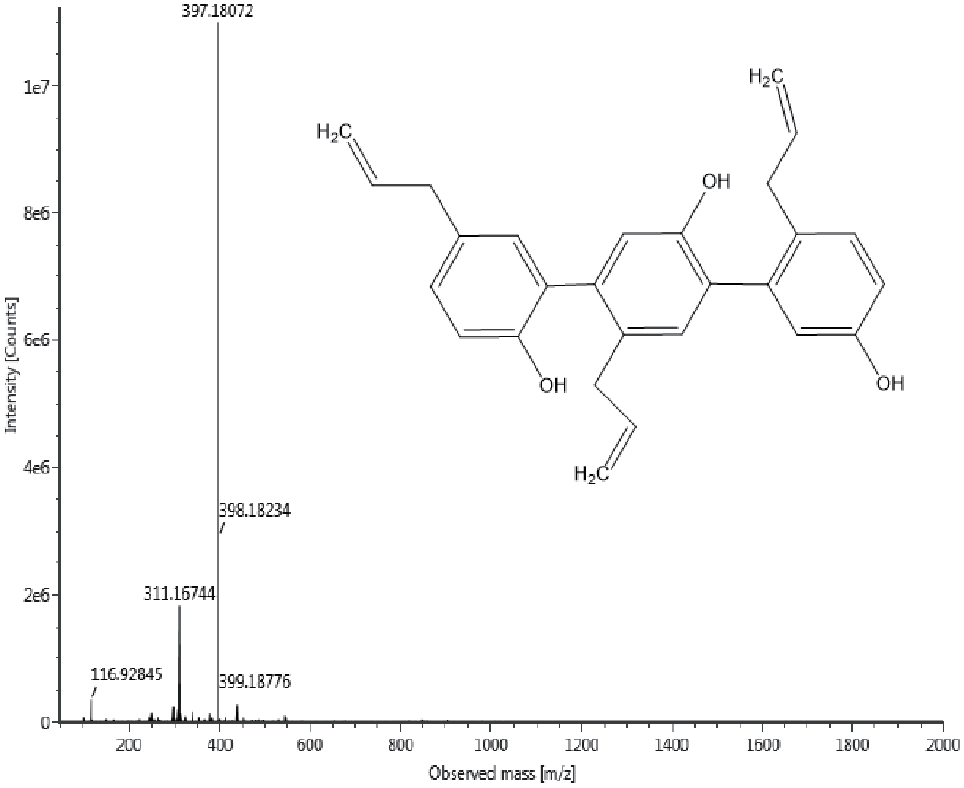

Figure S7 MS analysis of the by-product. Its Molecular formula was $C_{27}H_{26}O_3$ (RT= 13.8 min), hypothesized to be the trimer Dunnianol or its isomer, derived from chavicol.

A B

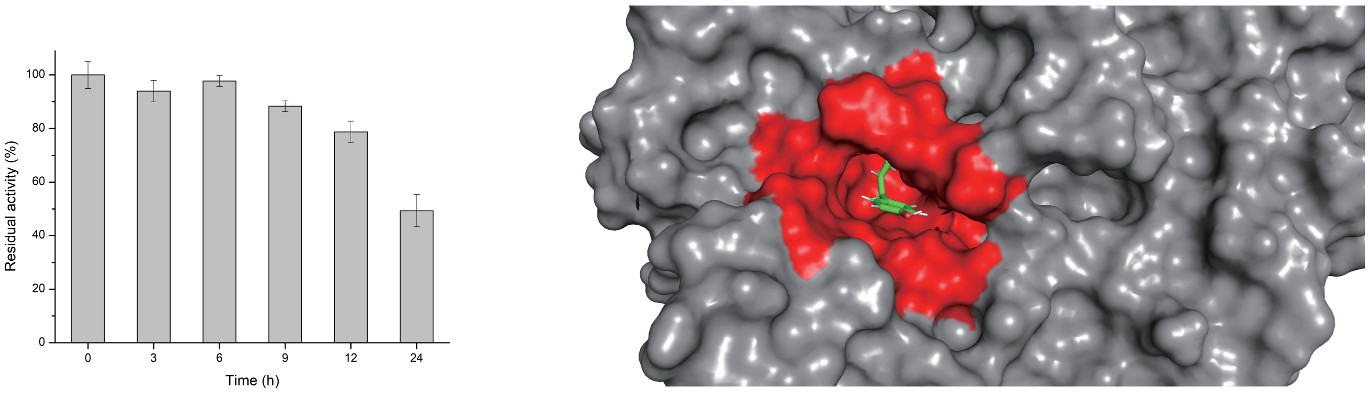

Figure S8 The influence of heat treatment time on thermal stability and Alanine scanning of residues within 5 Å of the substrate for MoLAC14.

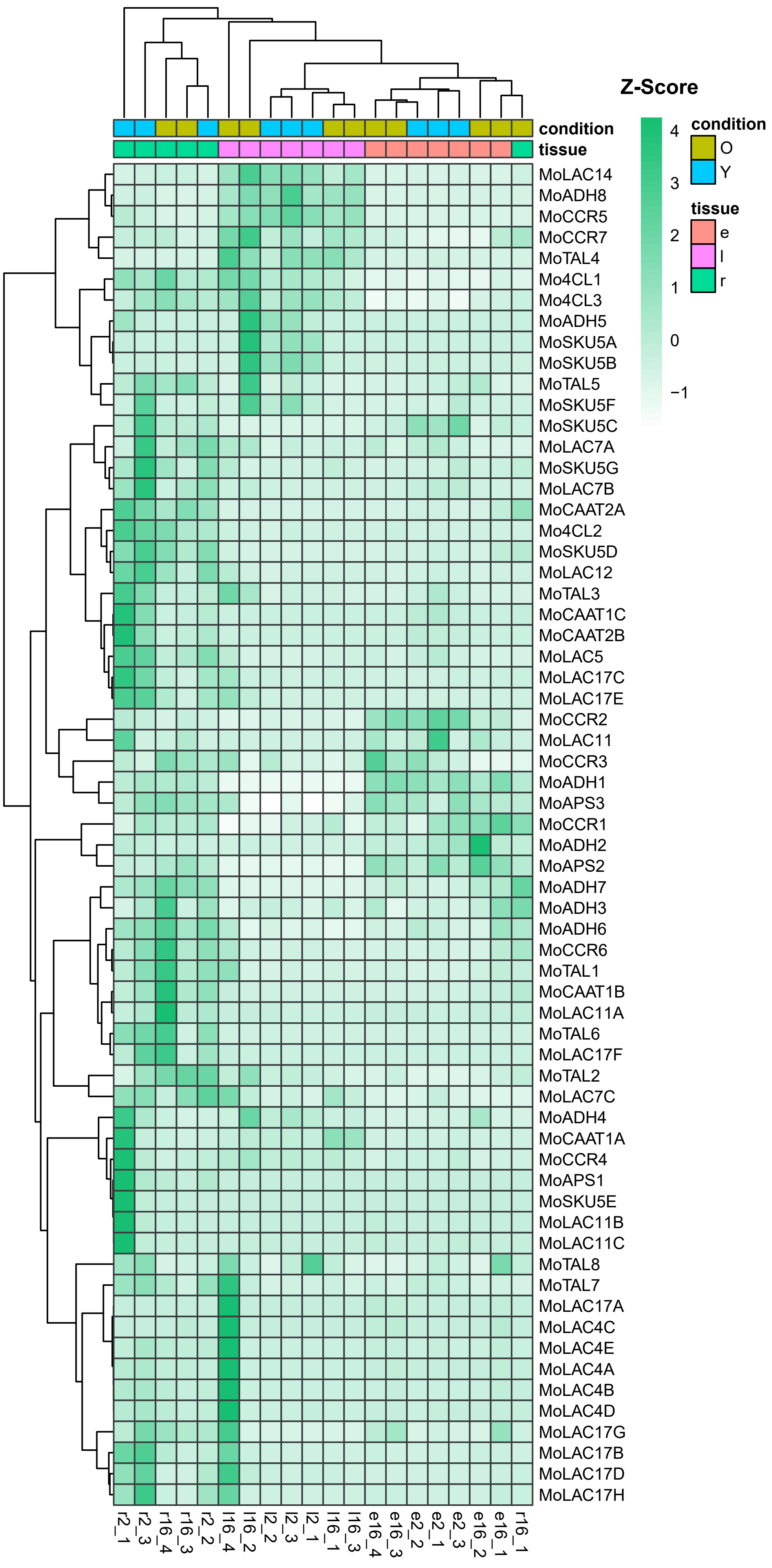

Figure S9 Genes with multiple copies involved in the synthesis from tyrosine to magnolol.
